# Self‐perceived preparedness for practice among graduating physical medicine & rehabilitation residents

**DOI:** 10.1002/pmrj.13246

**Published:** 2024-11-19

**Authors:** Nathan A. Wasserman, Laura Y. Huang, Diana M. Molinares, Timothy Tiu

**Affiliations:** ^1^ Department of Physical Medicine & Rehabilitation University of Miami Miller School of Medicine Miami USA

## Abstract

**Background:**

There is little research regarding the self‐perceived preparedness of residents to enter independent practice after training. Given the vast breadth of physical medicine & rehabilitation (PM&R) and the increasingly complex and wide‐ranging responsibilities and roles of physiatrists, this study is necessary to evaluate residents' perspectives of how programs are preparing them in the face of the changing practice environment.

**Objective:**

To identify how graduating PM&R residents perceived their training to prepare them for future practice. The researchers assessed perceived preparedness in six domains: (1) evaluation and management of conditions, (2) settings and responsibilities of practice, (3) familiarity with administrative processes, (4) physiatric‐specific prescriptions, (5) performing procedures, and (6) interpretation of diagnostic studies.

**Design:**

Survey.

**Setting:**

Virtual.

**Participants:**

Graduating PM&R residents in their final year of training in the United States were invited to complete the survey. Of 415 graduating residents, 54 accessed the survey, and 40 (9.6%) fully completed questions relating to preparation by residency.

**Interventions:**

Not applicable.

**Main Outcome Measure:**

Self‐perceived preparedness for practice among graduating residents across 70 subdomains of practice.

**Results:**

Mean preparedness was highest in the domain of physiatric prescription (3.45/5), and preparedness for administrative processes was significantly lower than all other domains (mean 2.25/5, *p* < .001). Across subdomains, the highest preparedness was in performing electromyography (4.48/5). Medical skills rated less than 2.50/5 included interpreting urodynamics (1.93/5), performing osteopathic manipulative therapy (1.57/5), and performing unguided peripheral nerve injections (2.25/5).

**Conclusions:**

Ultimately, residency programs should increase administrative training and identify internal strengths and weaknesses by polling their residents.

## INTRODUCTION

Physical medicine & rehabilitation (PM&R) is a broad and continually evolving field that has grown rapidly over the past decade.^1^ Conditions within the realm of physiatry affect all organ systems, with an age range from newborns to end of life. The responsibilities and roles of physiatrists in caring for these patients are increasingly complex and wide ranging, spanning from coordinating and implementing medical and rehabilitative care, performing and interpreting diagnostic examinations, conducting interventional procedures such as injections and percutaneous tenotomies, prescribing adaptive medical equipment, and advocating with government for persons living with disability—the largest unrecognized minority population.

Between the academic years of 2009–10 and 2019–20 there has been an approximately 12% increase in the number of graduating residents (Table 1).^2^, ^3^ When evaluating graduating residents during the 2004–05 academic year, the majority of residents did not believe a fellowship was necessary for any clinical area within physiatry except for pediatric rehabilitation.^4^ More recently, a study found that 81.1% of residents felt they were adequately prepared for general practice.^5^ Despite this, there has been an increasing trend for residents to pursue fellowship training,^4^, ^5^, ^6^, ^7^ with a recent study demonstrating that 73.7% of residents were planning on fellowship. Of these residents, 72.2% did not change their subspecialty focus during the course of training.^5^

**TABLE 1:** Physical medicine & rehabilitation residency statistics.^2^, ^3^

	2009–2010	2019–2020	% change
Number of programs	79	94	19.0
Total residents	1237	1453	17.5
Graduating residents	372	415	11.6

When ranking residencies, prospective PM&R candidates most heavily valued the perceived happiness of residents within a program, followed by opportunities for hands‐on procedures.^8^ The desire for procedural experience may be associated with the increasing trend for residents to pursue an interventional (pain medicine, sports medicine, or interventional spine) fellowship, which has increased from 54% in 2013–14 to 80% in 2019–20.^5^

There is little research regarding the self‐perceived preparedness of residents to enter independent practice after training. In the face of such a broad, heterogeneous field, many residency programs likely have numerous areas in which their training programs are not sufficiently preparing residents to practice independently, which may be due to factors such as resource availability or faculty areas of expertise. It is possible that as PM&R has grown increasingly complex, certain areas have inevitably been deemphasized in training and this has led to increased pursuit of fellowship training programs.

The goal of this study was to assess how graduating PM&R residents perceived their training to prepare them for practice in numerous domains and subdomains of PM&R.

## METHODS

### Data collection

An anonymous survey designed using Qualtrics software was emailed to PM&R residency program coordinators using publicly available contact information on the Accreditation Council for Graduate Medical Education (ACGME) website. The coordinators received an introductory letter and survey link and were requested to distribute them to senior residents. Residents were asked to rank their self‐perceived preparation for clinical practice across six overarching domains that were further broken down into 70 subdomains which residents ranked their preparedness in. The six domains were made with consideration of ACGME program requirements and author experience and were as follows: (1) evaluation and management of conditions, (2) settings and responsibilities of practice, (3) familiarity with administrative processes, (4) physiatric‐specific prescriptions, (5) performing procedures, and (6) interpretation of diagnostic studies. A 5‐point Likert scale was used; 1 = not well at all, 2 = slightly well, 3 = moderately well, 4 = very well, and 5 = extremely well. Respondents were asked to indicate demographic data, graduating class size, chief resident status, and postgraduation plans.

### Statistical methods

An analysis of variance (ANOVA) with post hoc Tukey analysis was performed for average readiness scores across the six survey domains. The average readiness score for a domain was defined as the overall mean of response scores across the subdomains that comprised one of the six larger domains.

Additionally, six ANOVAs with post hoc Tukey analysis were performed to compare the subdomains within each of the six survey domains.

Next, descriptive statistics were used to analyze postgraduation plans. Resident preparedness was then defined as the overall mean of response scores across the 70 subdomains assessed; a higher score meant a participant felt more prepared. A *t*‐test was performed to evaluate the difference between preparedness for residents entering fellowships and those entering unsupervised practice, as well as between those who served as chief residents and those who did not. One resident did not respond to the fellowship question and was excluded from this analysis only. A *t*‐test was also performed on the readiness scores of those entering practice in hospitals and those entering another form of practice, and an ANOVA was used to confirm equal variances between the two groups. For those entering fellowships, an ANOVA was performed on the readiness scores for each fellowship type, followed by a post hoc Tukey analysis.

Lastly, a linear regression was performed to analyze the impact of graduating class size on readiness scores. The one resident who reported a graduating class size of 24 was excluded from this analysis only as all the other class sizes were nine residents or less.

## RESULTS

Of 415 graduating residents, 54 accessed the survey, and 40 (9.6%) fully completed questions relating to preparation by residency. The 14 residents who did not respond to each question were excluded from the analysis. Most respondents came from either the South, Northeast, or Midwest regions, with only one respondent from the West region (Table 2).

**TABLE 2:** Geographic locations of survey respondents' residency programs.

Region	Number of respondents	Programs in region (2024)	Expected number of respondents	Actual to expected respondent ratio
Northeast	11	37	14	0.79
South	16	29	11	1.45
Midwest	12	26	10	1.2
West	1	13	5	0.20

The results for each of the six domains are found in Tables 3, 4, 5, 6, 7, 8. Overall, residents felt most prepared to evaluate and manage spine disorders, traumatic brain injury, and stroke, but felt underprepared for pulmonary, cardiac, and cancer rehabilitation. On average, residents reported being at least “very well” prepared for both inpatient and outpatient practice, but less than moderately well prepared for research, leadership roles, workers' compensation/disability care, and contract negotiation. Administratively, residents reported being less than “moderately well” prepared across all subdomains. In terms of prescriptions, residents were less than “moderately well” prepared for unsupervised home exercise programs, workplace modifications and restrictions, and modalities. Procedurally, residents were least prepared for osteopathic manipulative therapy (OMT), guided and nonguided peripheral nerve injections, non‐spine fluoroscopic injections, and diagnostic ultrasound (US). They felt most prepared with performing electromyography (EMG), nerve conduction studies (NCS), and spasticity procedures. Diagnostically, residents reported being least prepared with interpreting urodynamic studies and gait analysis. Across all subdomains, the highest preparedness was in performing EMGs (4.48/5) and the lowest was in performing OMT (1.58/5).

**TABLE 3:** How well has your residency prepared you for independently evaluating and managing the following conditions.

#	Field	Minimum	Maximum	Mean (n = 40)	SD
1	Spine disorders	3.00	5.00	4.28	0.71
2	Traumatic brain injury	3.00	5.00	4.20	0.75
3	Stroke	2.00	5.00	4.17	0.77
4	Spinal cord injury	2.00	5.00	4.08	0.82
5	Musculoskeletal injury (non‐spine)	1.00	5.00	3.98	1.08
6	Amputations	1.00	5.00	3.58	1.16
7	Neuromuscular disease (eg, muscular dystrophy, motor neuron disease)	1.00	5.00	3.15	1.06
8	Other medical rehabilitation (eg, transplant, lymphedema)	2.00	5.00	3.08	0.85
9	Other brain disorders (eg, Parkinson's, multiple sclerosis)	1.00	5.00	3.00	0.87
10	Pediatrics	1.00	5.00	3.00	1.24
11	Cancer rehabilitation	1.00	5.00	2.65	1.09
12	Cardiac rehabilitation	1.00	5.00	2.45	1.02
13	Pulmonary rehabilitation	1.00	5.00	2.35	0.99

**TABLE 4:** How well has your residency prepared you for the following settings and responsibilities of practice.

#	Field	Minimum	Maximum	Mean (n = 40)	SD
1	Acute inpatient rehabilitation	2.00	5.00	4.45	0.71
2	Inpatient consults	1.00	5.00	4.25	0.89
3	Outpatient clinic	1.00	5.00	4.20	0.98
4	Wellness and work/life balance	1.00	5.00	3.45	1.24
5	Billing and coding	1.00	5.00	3.02	1.17
6	Research (institutional review board approval, getting published, etc.)	1.00	5.00	2.80	0.95
7	Contract negotiation	1.00	5.00	2.25	0.99
8	Worker's compensation/disability	1.00	4.00	2.25	0.97
9	Leadership (eg, unit director, marketing, hiring staff)	1.00	5.00	2.05	1.18

**TABLE 5:** How well has your residency prepared you for the following administrative processes.

#	Field	Minimum	Maximum	Mean (n = 40)	SD
1	Peer‐to‐peer communication with insurance	1.00	5.00	2.85	1.44
2	Prior authorizations	1.00	5.00	2.58	1.39
3	RVUs	1.00	5.00	2.48	1.20
4	Billing vs. collections	1.00	5.00	1.90	1.20
5	Academic promotion (eg, assistant to associate professorship)	1.00	5.00	1.88	1.05
6	FTEs	1.00	5.00	1.80	0.95

Abbreviations: FTE, full‐time equivalent; RVU, relative value unit.

**TABLE 6:** How well has your residency prepared you for independently prescribing the following treatments.

#	Field	Minimum	Maximum	Mean (n = 40)	SD
1	EMG/NCS	2.00	5.00	4.38	0.76
2	Nonopioid analgesics	1.00	5.00	4.20	0.98
3	Referrals to surgical specialties	1.00	5.00	3.90	0.97
4	Radiology studies	1.00	5.00	3.85	1.06
5	Referrals to nonsurgical specialties	1.00	5.00	3.83	0.97
6	Antiseizure and Antispasmodics	2.00	5.00	3.80	0.84
7	Opioid analgesics	1.00	5.00	3.75	0.94
8	Physical therapy	1.00	5.00	3.65	1.24
9	Precautions (eg, fall, seizure, cardiac)	2.00	5.00	3.58	0.95
10	Prostheses	1.00	5.00	3.55	1.14
11	Occupational therapy	1.00	5.00	3.52	1.26
12	Other assistive devices (eg, walkers)	1.00	5.00	3.52	1.12
13	Psychopharmacologics	2.00	5.00	3.48	0.89
14	Other durable medical equipment or safety devices (eg, shower chair, grab bars)	1.00	5.00	3.45	1.14
15	Speech therapy	1.00	5.00	3.33	1.31
16	Nonspine orthoses	1.00	5.00	3.33	1.03
17	Neuropsychology evaluations	1.00	5.00	3.15	1.28
18	Wheelchairs/seating	1.00	5.00	3.15	1.17
19	Spine orthoses	1.00	5.00	3.13	1.05
20	Unsupervised home exercise program	1.00	5.00	2.98	1.21
21	Workplace modifications	1.00	5.00	2.70	1.19
22	Work restrictions (eg, lifting limit)	1.00	5.00	2.63	1.22
23	Modalities (eg, fluidotherapy, electrostimulation)	1.00	5.00	2.60	1.18

Abbreviations: EMG, electromyography; NCS, nerve conduction studies.

**TABLE 7:** How well has your residency prepared you for independently performing the following procedures.

#	Field	Minimum	Maximum	Mean (n = 40)	SD
1	Nerve conduction studies	3.00	5.00	4.53	0.63
2	Electromyography	3.00	5.00	4.47	0.71
3	Spasticity intramuscular injections, unguided	1.00	5.00	4.25	0.97
4	Spasticity intramuscular injections, guided	1.00	5.00	3.92	1.13
5	Musculoskeletal injections, unguided	1.00	5.00	3.88	1.08
6	Musculoskeletal injections, ultrasound guided	1.00	5.00	3.40	1.16
7	Spine injections, fluoroscopy guided	1.00	5.00	3.38	1.20
8	Diagnostic ultrasound	1.00	5.00	2.98	1.04
9	Musculoskeletal (nonspine) injections, fluoroscopy guided	1.00	5.00	2.92	1.29
10	Peripheral nerve injections, guided	1.00	5.00	2.83	1.32
11	Peripheral nerve injections, unguided	1.00	5.00	2.25	1.28
12	Osteopathic manipulative therapy (OMT)	1.00	5.00	1.57	1.09

**TABLE 8:** How well has your residency prepared you for independently interpreting the following diagnostic studies.

#	Field	Minimum	Maximum	Mean (n = 40)	SD
1	Nerve conduction studies	1.00	5.00	4.17	0.86
2	Electromyography	1.00	5.00	4.17	0.92
3	X‐ray	1.00	5.00	3.40	0.97
4	CT/MRI	1.00	5.00	3.20	1.08
5	Ultrasound	1.00	5.00	3.05	1.02
6	Gait analysis	1.00	5.00	2.95	1.00
7	Urodynamics	1.00	4.00	1.93	0.91

Abbreviations: CT, computed tomography; MRI, magnetic resonance imaging.

There was a significant difference between the average preparation scores reported in the familiarity with administrative processes domain and the score reported in each of the five other domains (all *p* < .001), though there was no significant difference between the scores for any of the other domains (Table 9).

**TABLE 9:** Average self‐reported preparedness scores for different competency domains upon completion of physical medicine & rehabilitation residency.^a^

#	Competency domain	n	Mean readiness score	SD
1	Evaluation and management of conditions	13	3.38	0.70
2	Settings and responsibilities of practice	9	3.19	0.94
3	Familiarity with administrative processes	6	2.25	0.44
4	Prescription of treatments	23	3.45	0.46
5	Performance of procedures	12	3.37	0.91
6	Interpretation of diagnostic studies	7	3.27	0.77

^a^ Mean readiness scores are overall means of Tables 3, 4, 5, 6, 7, 8, respectively. Each subdomain was weighted equally to calculate the domain mean.

**FIGURE 1:**
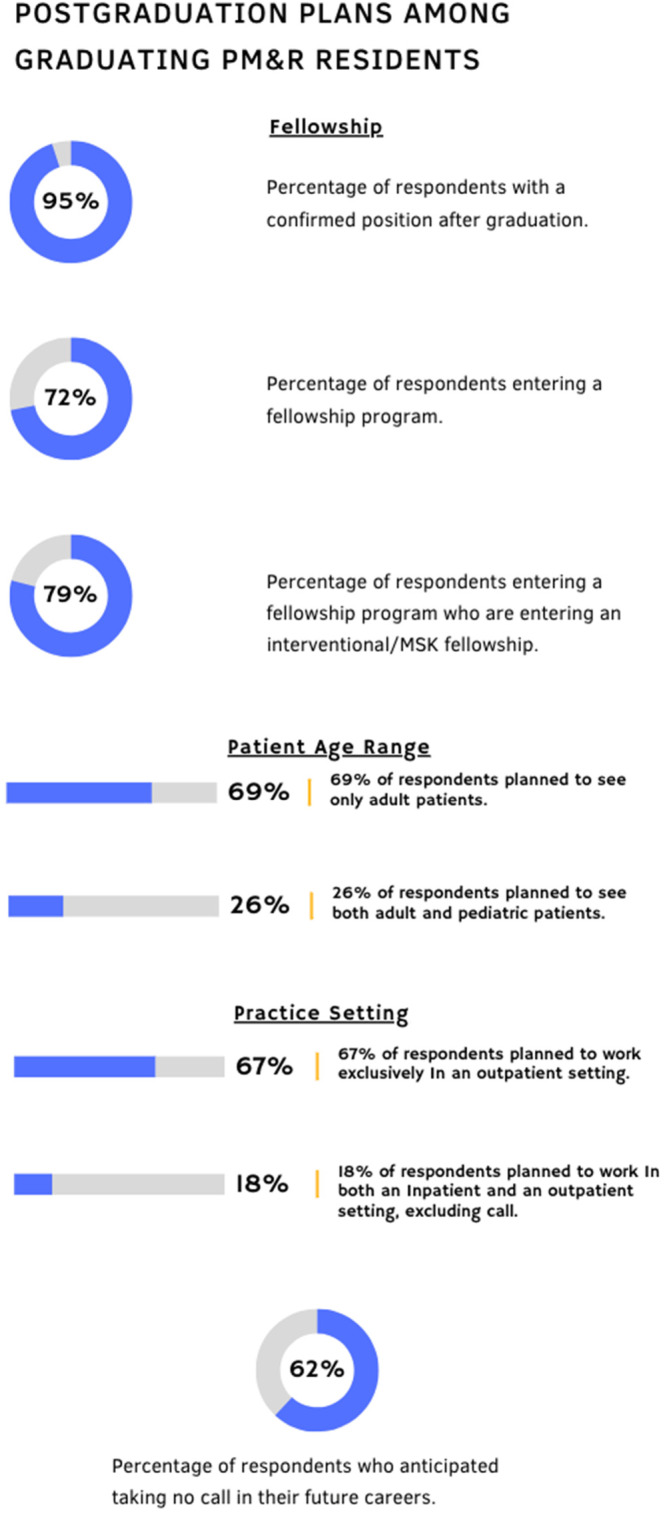
Postgraduate plans of graduating physical medicine & rehabilitation residents. MSK, musculoskeletal; PM&R, physical medicine & rehabilitation.

Of all respondents, 95% had a confirmed position for after graduation, with 72% entering a fellowship program. Of the fellowship programs, 79% were in pain medicine, sports medicine, or a non‐ACGME sports and spine program. Of the graduates, 69% planned on seeing only adult patients, and 26% expected to see a combination of an adult and pediatric population. When asked about their anticipated setting of practice, 67% of respondents expected to work exclusively in an outpatient setting, and 18% expected to work in a combination of inpatient and outpatient, excluding call. 62% of graduating residents did not anticipate taking any call in their future careers (Figure 1). All graduating residents planned on performing procedures of some kind in their practices, with the most common being non‐guided and US‐guided musculoskeletal injections, followed by guided perineural injections.

The 28 residents entering a fellowship felt significantly less prepared by residency as a whole (*M* = 3.08, *SD* = 0.62) than the 11 residents entering unsupervised practice (*M* = 3.81, *SD* = 0.61), *t*(37) = 2.09, *p* < .01. Among the residents entering a fellowship, the two entering a brain injury medicine fellowship (*M* = 1.77, *SD* = 0.16) felt they were significantly less prepared by residency than the four entering non‐ACGME sports/spine (*M* = 3.20, *SD* = 0.59), *p* < .05, the five entering pain medicine (*M* = 3.29, *SD* = 0.62), *p* < .05, and the 13 entering sports medicine (*M* = 3.15, *SD* = 0.45), *p* < .05. Among the residents not entering a fellowship, the six beginning work at a hospital (*M* = 4.11, *SD* = 0.13) felt better prepared by residency than the five beginning work elsewhere (*M* = 3.44, *SD* = 0.77), though this result was not significant *t* (9) = 2.26, *p* = .060.

**FIGURE 2:**
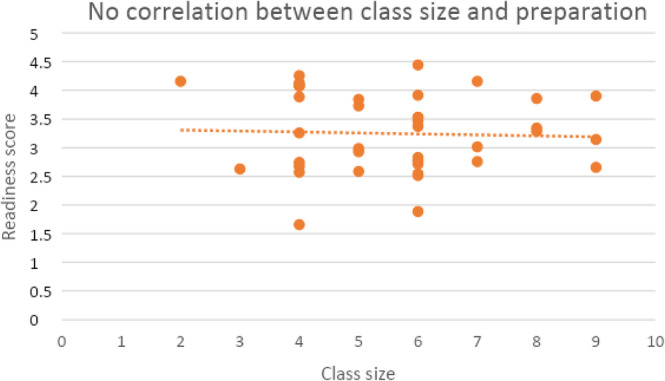
A regression analysis showed no correlation (R = 0.041, *p* = .803) between physical medicine & rehabilitation self‐reported preparation by residency upon graduation and size of graduating class.

There was no difference in perceived preparation between the 21 chief residents (*M* = 3.29, *SD* = 0.69) and the 19 non‐chief residents (*M* = 3.24, *SD* = 0.71), *t*(38) = 2.02, *p* = .834. Additionally, a regression analysis showed no difference in preparation associated with residency graduating class size (R = 0.041, *p* = .803) (Figure 2).

## DISCUSSION

Graduating residents felt less prepared for rehabilitation in non‐ACGME subspecialties such as cancer, pulmonary, cardiac, and transplant rehabilitation, as well as pediatric rehabilitation. Pediatrics has been a subspecialty in which fellowship pursual predates the current 2‐decades long boon, with the previously mentioned 2004 study that found residents felt fellowship was necessary only for pediatric rehabilitation.^4^ However, the growth of other non‐ACGME subspecialties that may not be taught as much during residency can explain some of the growth of noninterventional fellowship pursual. Cancer rehabilitation, in particular, has grown since the 2004 study, with the first such fellowship program opening in 2007.^9^

Among all large domains, residents ranked their familiarity with administrative processes significantly lower than their preparedness in all other domains. Among aspects of practice, research and leadership also notably ranked low. Given the significant burden of administrative duties, educators should take responsibility to better prepare trainees for nonclinical aspects of medicine. Physiatrists have been shown to have high levels of burnout, with 48%–62% of all practicing specialists showing signs of burnout.^10^ Regulatory demands have consistently been rated as a top reason for burnout, and poor preparation for meeting these demands in residency may be contributing to the stress on practicing physiatrists.^11^, ^12^, ^13^

Another notable clinical finding of this study was the relatively low preparedness rankings in ultrasound (US), both as a means to guide injections and as a diagnostic tool. Residents ranked their preparedness for unguided musculoskeletal and spasticity injections higher than their preparedness in guided injections (3.88/5 to 3.4/5). Research has consistently demonstrated that US‐guided musculoskeletal injections have superior outcomes and has strongly suggested that EMG‐ or US‐guided spasticity injections are superior to landmark guided injections.^14^, ^15^ However, as unguided injections are typically more straightforward to perform this result is not wholly surprising and may not truly reflect an inability of graduating residents to perform guided injections.

Residents also ranked their preparedness to interpret and perform diagnostic US below their ability to interpret EMG/NCS, x‐ray, computed tomography, and magnetic resonance imaging. Although imaging tools have their place in physiatry practice, US can be done at bedside to make rapid diagnoses and limit the need for excess follow‐up appointments, costly advanced imaging modalities, and unnecessary radiation exposure. It is, however, encouraging to see that graduating physiatrists ranked their readiness at least 3/5 in each of the imaging modalities mentioned, indicating an ability to incorporate each into their practice to a degree when necessary or otherwise continue training with them in fellowship.

Given these results, it is perhaps not surprising that 79% of graduates planning for fellowship (and 57% of graduates overall) intended to pursue some interventional musculoskeletal fellowship (pain medicine, sports medicine, or non‐ACGME interventional spine and musculoskeletal medicine). The finding that residents entering fellowship felt significantly less prepared than residents entering unsupervised practice adds to the notion that the increase in fellowship pursual over the past 2 decades may be due to residents feeling underprepared for the interventional careers they are increasingly seeking.^4^, ^5^, ^6^, ^7^ As EMG/NCS teaching during residency has largely been successful in preparing residents, increasing US and/or fluoroscopy requirements to be similar to EMG/NCS requirements may be a next step to address this changing landscape.

Notably, among residents not entering a fellowship, those beginning hospital employment felt better prepared by residency than those working elsewhere, though the result was not significant (*p* = .06). It is possible that those who had better experiences in their residency programs, which are largely based in a hospital environment, would be more likely to pursue employment in that environment.

### Limitations

The chief limitation of the study was the low response rate, with 40 of 415 graduating residents (9.6%) fully completing the survey. As a result, many programs were altogether not included in the results. Additionally, the low response rate may have introduced selection bias into the study as people who felt their training was inadequate may have been more likely to respond in an effort to increase the quality of training for future residents. Despite this, the authors believe the study adequately serves as a starting point for residency programs to introspect. It can be used as a comparison tool to determine a program's own strengths and weaknesses, with the caveat that small differences in mean readiness scores are not likely to be true differences. Individual programs should be wary of ignoring large differences between their program and this study due to lack of statistically significant differences as comparisons of this nature are likely to be statistically underpowered. Ultimately each program will have to combine their own best judgment with the data collected.

The low response rate also led to insufficient power to measure relationships between future career plans (eg, procedure‐focused practice) and pursuit of fellowship. Future research can investigate this and other trends.

Additionally, the study was geographically limited as there was only one respondent from any program on the west coast, so results may not apply to programs in that area. Although other regions were not represented exactly proportionally with the number of programs available in each region, their proportional representation ranged from 0.79–1.45, which would have been expected. With this being said, programs on the west coast could still use these methods as a guide to assess their own program in comparison to the rest of the country.

As the study measures self‐perceived preparedness rather than objectively measured preparedness, it is also subject to a degree of recall bias. Responses may be influenced by factors other than training quality and respondents may not remember aspects of their training in certain areas. However, it is not feasible to objectively measure preparedness in 70 subdomains, and the 5‐point Likert scale used has been well validated.^16^

### Conclusion

PM&R residents across the country feel confident in their training in certain areas, such as caring for patients with stroke, traumatic brain injury, and spinal cord injury, and performing and interpreting EMG/NCS, and less confident in other areas such as cardiac and cancer rehabilitation, US, fluoroscopy, research, performing administrative duties, leadership roles, and nonneurological rehabilitation. These latter activities are less emphasized in most residency programs.

Residency programs can bolster their educational programs and resident preparedness by annually assessing internal strengths and weaknesses collected by way of graduating resident surveys using the framework provided. This continual feedback loop will enable programs to target specific domains or subdomains in need of programs and those with relative success in these areas can consider sharing key aspects of their curriculum at academic conferences. Given that administrative learning among residents appears to be low across all institutions, programs should collaborate to create a new framework to improve teaching the nonclinical aspects of practice.

These data have the potential to help grow the field of medical education research and utilize evidence‐based methods to help national organizations and medical educators refine residency training programs. Future research should assess trends in postgraduate plans, efficacy of curriculum changes, and follow‐up surveys to recent graduates including questions on which areas they would have liked more training in. The role of fellowships in the education of PM&R residents needs further assessment as well.

## DISCLOSURES

None.
